# Cytokine Patterns in Tuberculosis Infection; IL-1ra, IL-2 and IP-10 Differentiate Borderline QuantiFERON-TB Samples from Uninfected Controls

**DOI:** 10.1371/journal.pone.0163848

**Published:** 2016-09-29

**Authors:** Ida Wergeland, Jörg Assmus, Anne Ma Dyrhol-Riise

**Affiliations:** 1 Department of Clinical Science, Faculty of Medicine and Dentistry, University of Bergen, N-5020 Bergen, Norway; 2 Center for Clinical Research, Haukeland University Hospital, N-5020 Bergen, Norway; 3 Department of Infectious Diseases, Oslo University Hospital, Oslo, Norway; 4 Institute of Clinical Medicine and K.G. Jebsen IRC, Faculty of Medicine, University of Oslo, Oslo, Norway; University of Cape Town Lung Institute, SOUTH AFRICA

## Abstract

**Background:**

Interferon gamma release assays (IGRAs) do not discriminate between active tuberculosis (TB) and latent TB infection (LTBI), which limit their use in TB endemic areas. Subjects with QuantiFERON-TB (QFT) results around the diagnostic cut-off more likely show inconsistent results on serial testing which makes the interpretation of the assay difficult. We have studied potential biomarkers in patients with various stages of TB infection and with borderline QFT tests compared to those with higher values.

**Methods:**

27 soluble biomarkers were analysed in QFT supernatants from patients with active TB (n = 18), individuals with LTBI (n = 48) and from QFT negative controls (n = 16) by the Multiplex bead assay. The LTBI group was classified into two groups according to QFT IFN-γ levels; QFT borderline (0.35–0.70 IU/mL, n = 11) or QFT high (>0.70 IU/mL, n = 36).

**Results:**

The levels of IL-1ra, IL-2, IL-13, IL-15, IFN-γ, IP-10 and MCP-1 in background corrected TB antigen stimulated supernatants (TBAg-Nil) significantly distinguished both active TB and LTBI QFT high groups from the QFT negative controls (p≤0.004). In addition, IL-1ra, IL-2 and IP-10 significantly differentiated the QFT borderline group from the controls (p≤0.001). Still, in the QFT borderline group the IL-1ra and IP-10 levels were not significant different from neither the QFT high nor the active TB group, whereas the IL-2 levels were lower (p≤0.003). The level of IP-10 showed the best separation between the QFT borderline group and the QFT negative controls (AUC 0.92) and offered 100% sensitivity for active TB.

**Conclusion:**

IL-1ra, IL-2 and IP-10 differentiate QFT borderline samples from uninfected controls and the majority of QFT borderline subjects were classified as LTBI by these markers. Still, inconsistency was seen, and further studies are needed to examine the performance of alternative markers before concluded if they could be used as diagnostics tools.

## Introduction

Screening for latent tuberculosis infection (LTBI) has traditionally been performed by the tuberculin skin test (TST), but during the last years interferon gamma (IFN-γ) release assays (IGRAs) have been increasingly used for this purpose. IGRAs offer better specificity than the TST, but have suboptimal sensitivity in children and human immunodeficiency virus (HIV) co-infected individuals, and the tests cannot distinguish between active tuberculosis (TB) and LTBI [1]. This limits their use in high TB incidence areas where the burden of LTBI and HIV co-infection is high. In addition, studies of IGRAs for serial TB screening of health care workers (HCW) [2,3] and in HIV infection [4,5] have shown that the interpretation of IGRA results is complicated by relative high rates of conversions and reversions and within subject variability. Subjects with QuantiFERON-TB (QFT) baseline results around the diagnostic cut off are more likely to have inconsistent results on serial testing, and a systematic review suggests introduction of a borderline zone for QFT when screening HCW [2]. It has also been shown that there is substantial variability in QFT response when the test is repeated in the same patient sample [6]. As strategies for TB control and elimination include preventive therapy to individuals with LTBI to reduce the risk for development of active disease, there is a need of more robust and reliable diagnostic tools which can differentiate TB infected from non-infected individuals.

Several studies aiming to identify alternative biomarkers for use in TB diagnostics have been conducted. IFN-γ inducible protein 10 (IP-10) is the most studied soluble biomarker, and a review by Ruhwald *et al* concludes that whereas IP-10 based tests perform comparably to QFT in most patient groups, it seems to be more robust in children and HIV-infected individuals [7]. However, IP-10 has not been studied for use in serial testing. Further, although several markers or combination of markers have been suggested that may differentiate between the various stages of TB infection [8–12], no obvious candidate has been identified [13].

The aims of this study were to examine the potential of 27 different soluble markers detected in QFT supernatants to differentiate between the various stages of TB infection and to compare the pattern of markers in subjects with QFT test results in the borderline zone with those with higher values as well as with QFT negative controls. We show that the background corrected TB antigen stimulated levels (TBAg-Nil) of seven soluble markers (IL-1ra, IL-2, IL-13, IL-15, IFN-γ, IP-10 and macrophage chemoattractant protein 1 (MCP-1)) distinguished both the active TB and LTBI groups from the QFT negative controls. The level of IL-1ra, IL-2 and IP-10 also differentiated the QFT borderline group from the controls, supporting true TB infection in the majority of these patients.

## Material and Methods

### Study participants

Persons referred to the TB clinic at Haukeland University Hospital, Bergen, Norway for QFT testing and medical evaluation of LTBI based on known exposure of TB or origin from a high TB endemic country with a concomitant positive TST and patients diagnosed with active TB admitted to the inpatient ward, were included in a study of the performance of IGRA in clinical practice [14].

The diagnosis of active TB was based on a positive *Mycobacterium tuberculosis (Mtb)* culture or on clinical and radiological findings. Subjects with no signs of active TB based on X-ray, sputum or biopsy examination and clinical evaluation, and with a positive QFT test were defined as LTBI and offered preventive anti-TB chemotherapy with isoniazid and rifampicin for 3 months. A repetitive QFT test was performed during the first year after preventive therapy was ended. None of the study participants were HIV infected. The LTBI group were further classified according to QFT values 0.35–0.70 IU/mL (QFT borderline) or > 0.70 IU/mL (QFT high).

Written informed consent was obtained from all participants. The study was approved by the Regional Committee for Ethics in Medical Research (REK-Vest, 3.2005.823), Norway.

### QuantiFERON-TB GOLD in-tube assay

The assay was performed according to the manufacturer’s instructions (Cellestis Ltd, Qiagen, Chadstone, VIC, Australia). One ml of whole blood was added to each of the three QFT tubes containing TB antigen (ESAT-6, CFP-10 and TB 7.7), mitogen-positive control (phytohemagglutinin (PHA)) and a negative control, respectively. The tubes were incubated at 37°C for 16–24 h, centrifuged and the supernatant removed. The amount of IFN-γ in the supernatant was quantified by enzyme-linked immunosorbent assay (ELISA). The level of IFN-γ in the TB antigen (TBAg) tube was corrected for background by subtracting the IU⁄ml value obtained for the respective negative control tube (Nil). The cut-off value for positive test (TBAg-Nil) was ≥ 0.35 IU⁄ml. The excess of supernatants from the QFT tubes were frozen and stored at -80°C until further analysis.

### Multiplex cytokine analysis

Levels of biomarkers in QFT supernatants were measured using Bio-Plex Pro Human Cytokine Group 27-Plex Panel (Bio-Rad Laboratories Inc., Hercules, CA) on a Luminex 100 platform according to the manufacturer’s instructions. Levels of interleukin (IL)-1β, IL-1 receptor antagonist (IL-1ra), IL-2, IL-4, IL-5, IL-6, IL-7, IL-8, IL-9, IL-10, IL-12 (p70), IL-13, IL-15, IL-17, basic fibroblast growth factor (basic FGF), eotaxin, granulocyte colony stimulating factor (G-CSF), granulocyte macrophage colony stimulating factor (GM-CSF), IFN-γ, IP-10, MCP-1, macrophage inflammatory protein 1 alpha (MIP-1α), MIP-1β, platelet-derived growth factor -BB (PDGF-BB), regulated on activation, normal T cell expressed and secreted (RANTES), tumor necrosis factor-α (TNF-α) and vascular endothelial growth factor (VEGF) were analysed in supernatants both from the unstimulated (Nil) and the TB antigen stimulated QFT tubes (TBAg). Supernatants from the mitogen stimulated QFT tubes were not analysed. The supernatants were diluted 1:4 in Bio-Plex sample diluent, and levels obtained were therefore multiplied by four to correct for the dilution. STarStation v.3 software was used for data analysis.

For three of the 27 markers analysed (IL-8, MIP-1β and RANTES), a considerable proportion of the study subjects in both the active TB and LTBI groups had both TBAg and Nil levels that were above the upper detection limit (UDL) of the assay despite dilutions. Thus, these TBAg-Nil results were excluded from all statistical analyses. This pattern was also found for IP-10 and MCP-1, but only for the TBAg samples. Thus, the TBAg-Nil results of IP-10 and MCP-1 were not used in statistical analyses when the active TB and LTBI groups were compared, whereas when comparing the two TB infection groups with the controls the TBAg levels above the UDL were replaced by the highest value of the standard curve for the respective marker to allow non-parametric statistical analysis. Thus, when comparing the level of some markers both within the TB infection groups and with the controls, significant differences may not be detected because the actual median levels in the active TB and LTBI group may be higher than we were able to detect within the range of the assay. Also occasional values (<15% of the total data set) above the UDL for some of the other markers (IL-1β, IL-6 and MIP-1α), were replaced by the highest value of the standard curve. The level of IL-5 was below the lower detection level (LDL) of the assay in >75% of all samples, and were therefore excluded from all statistical analysis. For the other markers, the occasional values (<10% of the total data set) below the LDL were replaced by a defined common value below the LDL (0.001), which allowed non-parametric statistical analysis.

### Statistical analysis

All statistical analyses were performed using IBM SPSS statistics 21 and GraphPad Prism 6. The unstimulated level (Nil) and the background corrected TB stimulated level (TBAg-Nil) of each marker were included as separate variables in the statistical analyses.

Mann-Whitney U test was used to detect differences between the study groups. Wilcoxon matched pairs signed rank test was used to detect differences in the level of markers before and after prophylactic anti-TB chemotherapy. The general significance level was set to 0.05. In a preliminary step, the Spearman correlations between all pairs of biomarkers were calculated, showing a high proportion of correlated variables. Taking into account multiple testing effects the Bonferroni adjustment would be too conservative due to the dependence between the markers. Thus, we decided to use a marginal level of 0.005 (corresponding to Bonferroni adjustment for 10 tests).

Receiver operator characteristic (ROC) curve analyses were performed for the markers which, by the aforementioned tests, significantly differentiated between the LTBI borderline group and the QFT negative group. The optimal cut-off levels were defined by the minimum Euclidian distance to maximal specificity and sensitivity.

## Results

### Study participants

A total of 82 study participants had stored QFT supernatants available for Multiplex analysis, and these were classified into three groups; 1) active TB (n = 18), 2) QFT positive LTBI (n = 48), and 3) QFT negative controls (n = 16). Eleven of the QFT positive LTBI individuals were further classified as QFT borderline, 36 as QFT high and in one IFN-γ level was not determined. Fifteen individuals with LTBI had available repetitive samples obtained during the first year after preventive therapy. The clinical characteristics of the study participants are summarized in Table 1.

**Table 1:** Clinical characteristics of the study participants.

	Active TB, (n = 18)	LTBI QFT high (n = 36)^*^	LTBI QFT borderline (n = 11)^*^	QFT negative (n = 16)
Age; median (range)	32 (18–62)	40 (13–67)	40 (25–53)	47 (16–68)
Sex; males/females	6/12	13/23	4/7	8/8
Origin; TB high/low endemic country	16/2	22/14	9/2	4/12

LTBI QFT borderline = 0.35–0.70 IU/ml. LTBI QFT high >0.70 IU/mL.

*In total, n = 48 QFT positive individuals with LTBI were included in the study, but for one them the exact value of the QFT-test was not known. This subject was therefore excluded from the subgroup analyses.

The diagnosis of active TB was based on a positive *Mtb* culture in 16 of the patients, whereas in two patients the diagnosis was based on clinical and radiological findings. Fourteen patients had pulmonary TB and four extrapulmonary TB, and there were no multi- or extensively drug-resistant TB cases.

### Cytokine patterns in latent and active TB infection

**Fig 1:**
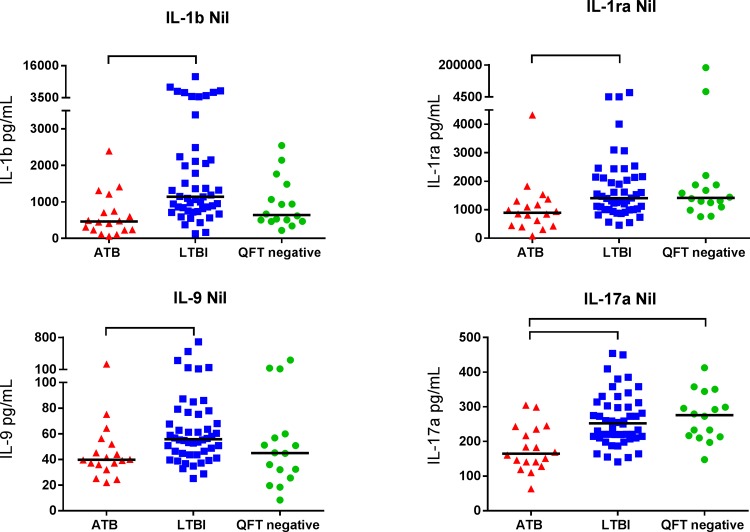
Markers differentiating between active and latent TB infection. Nil levels (pg/mL) of IL-1β, IL-1ra, IL-9 and IL-17a in patients with active TB (ATB), latent TB infection (LTBI) and in QFT negative controls (QFT negative). The horizontal lines show the median values. Mann-Whitney U test was used for comparison between groups. Brackets represents statistically significant differences (p≤0.004).

Twenty-seven different cytokines, including chemokines and markers of inflammation, were analysed in QFT supernatants and compared in patients with various stages of TB infection and with QFT negative controls. We first examined if there were any differences between patients with active TB and LTBI. For the markers within the range of the assay, we found no significant differences in TBAg-Nil levels between the active and the LTBI group. In contrast the Nil levels of IL-1β, IL-1ra, IL-9 and IL-17a were significantly lower in the active TB group compared with the LTBI group (p≤0.004, Fig 1).

**Fig 2:**
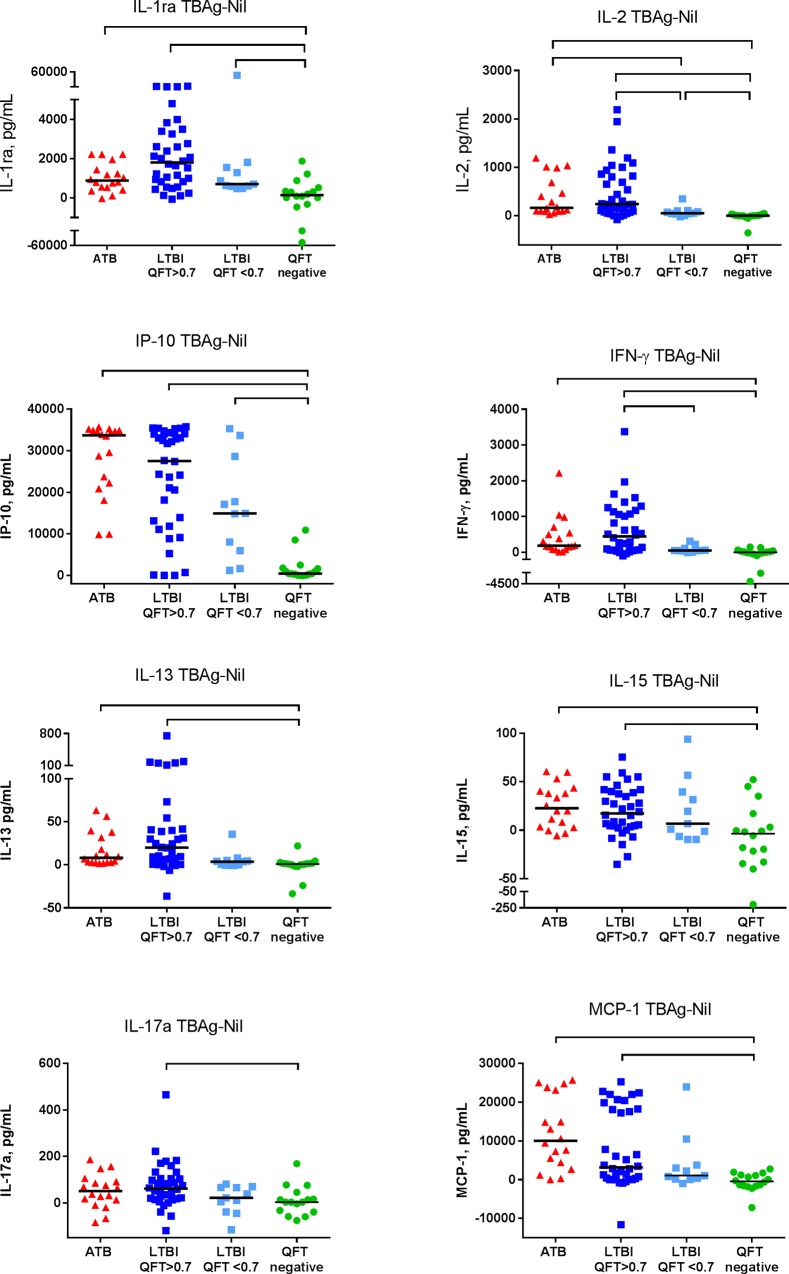
Markers differentiating TB infection from QFT negative controls. TB antigen stimulated background corrected (TBAg-Nil) levels of IL-1ra, IL-2, IP-10, IFN-γ, IL-13, IL-15, IL-17a and MCP-1 in patients with active TB (ATB), latent TB infected subjects with QFT result >0.70 IU/mL (LTBI QFT>0.7), subjects with QFT result in the borderline zone 0.35–0.70 IU/mL (LTBI QFT<0.7) and in QFT negative controls (QFT negative). The horizontal lines show the median values. Mann-Whitney U test was used for comparison between groups. The TBAg-Nil level of IP-10 and MCP-1 were excluded from statistical analyses when the LTBI and ATB groups were compared because high proportions of study subjects in both groups had levels above the UDL of the assay. Brackets represents statistically significant differences (p<0.005).

We then analysed if any marker could distinguish between TB infection and QFT negative controls. We found that the TBAg-Nil levels of seven of the markers; IL-1ra, IL-2, IL-13, IL-15, IFN-γ, IP-10 and MCP-1, were significantly higher in both the active TB and LTBI group than in QFT negative controls (p≤0.004) (Fig 2).

In the unstimulated supernatants, the Nil level of IL-15, eotaxin and basic FGF were significantly lower (p<0.0002) and the Nil level of RANTES significantly higher (p = 0.0003) both in the active TB and LTBI groups compared with the QFT negative controls. The median Nil levels of several other markers (IL-2, IL-4, IL-13, IL-17a and IFN-γ) were also lower in the active TB (p≤0.003) group than in controls (data not shown).

Fifteen of the study participants in the LTBI group (13/15 QFT high) had available supernatants from QFT tests performed during the first year after prophylactic anti-TB chemotherapy. There were no significant changes in the Nil or TBAg-Nil levels for any of the markers in response to treatment, except from a decline in the Nil level of PDGF-BB (p = 0.002). Also the TBAg-Nil level of IL-2, IFN-γ and IP-10 remained significantly higher than that seen in the QFT negative controls indicating a maintained response to TB antigens (data not shown).

#### Cytokine patterns in subjects with borderline zone QFT responses

Finally, we investigated if LTBI patients with borderline zone QFT IFN-γ values in the range 0.35–0.70 IU/ml, close to cut-off for the QFT test, demonstrated a different cytokine pattern than LTBI patients with higher QFT IFN-γ values > 0.70 IU/ml. The TBAg-Nil levels of IL-1ra, IL-2, IL-13, IL-15, IFN-γ, IP-10, MCP-1 and IL-17a were all significantly higher in the QFT high group than in the QFT negative controls (p≤0.004, Fig 2).

However, only the levels of IL-1ra, IL-2 and IP-10 significantly differentiated the QFT borderline group from the QFT negative controls (p≤0.001) (Fig 2). Whereas the IL-1ra and IP-10 levels in the QFT borderline group were not significant different from neither the QFT high nor the active TB group, the IL-2 level was significantly lower in the QFT borderline group (p≤0.003). In the Nil supernatants, only IL-15 and basic FGF significantly differentiated the QFT borderline group (lower levels) from the QFT negative controls (p≤0.0008), whereas for the QFT high LTBI group a similar pattern as seen for the overall LTBI group was found (data not shown).

The diagnostic accuracy of the TBAg-Nil levels of IL-1ra, IL-2 and IP-10 in differentiating between the QFT borderline group and the QFT negative group were investigated by ROC curve analyses. The AUC, p-value, optimal cut-off levels, sensitivity and specificity for the respective markers are given in Table 2. The TBAg-Nil level of IP-10 had the highest AUC (0.92).

**Table 2:** ROC curve analyses for differentiation between the QFT borderline group and QFT negative controls.

Marker	Cut-off (pg/mL)	Sensitivity, % (95% CI)	Specificity, % (95% CI)	AUC (95% CI)	p-value
IL-1ra (TBAg-Nil)	409	100 (72–100)	75(48–93)	0.852 (0.701–1.000)	0.002
IL-2 (TBAg-Nil)	37	82 (48–98)	94 (50–100)	0.875 (0.720–1.000)	0.001
IP-10 (TBAg-Nil)	4235	82 (48–98)	88 (62–98)	0.920 (0.821–1.000)	<0.001

Based on the cut-off levels found by the ROC curve analyses, four subjects classified as LTBI by the QFT test, two in the QFT borderline group and two in the QFT high group, were classified as not TB infected by IP-10, and two subjects classified as negative by the QFT test were classified as LTBI (Table 3). IP-10 had a 100% sensitivity for active TB. IL-1ra and IL-2 classified four of the subjects in the QFT high group as not TB infected, and IL-2 also classified two subjects in the QFT borderline group as not infected. In the QFT negative control group, four and one subjects, respectively, were classified as LTBI by IL-1ra and IL-2. In total, 18/81 of the study subjects were classified in discordance with the QFT test by one or several of the three alternative markers. For eleven subjects only one of the markers showed a discordant result, whereas for seven subjects two or all three of the alternative markers were discordant with the QFT classification. In the QFT high group, four of the seven subjects that were classified in discordance with the QFT test by one or several of the three alternative markers, had low QFT test values between 0.70–1.0 IU⁄mL.

**Table 3:** Number of subjects classified as active TB and LTBI by IL-1ra, IL-2 and IP-10.

Marker	Active TB (n = 18)	LTBI QFT high (n = 36)	LTBI QFT borderline (n = 11)	QFT negative (n = 16)
IL-1ra	14	32	11	4
Il-2	17	32	9	1
IP-10	18	32*	9	2

*Two subjects in the LTBI QFT high group had an inconclusive IP-10 TBAg-Nil value because both the TBAg and the Nil value were out of range of the assay.

## Discussion

We have examined the potential of several soluble chemokines and cytokines measured in QFT supernatants to diagnose and differentiate between the various stages of TB infection and uninfected controls as well as the pattern of markers in subjects with QFT test result in the borderline zone of 0.35–0.70 IU/mL. We found that the background corrected TB antigen stimulated levels of seven markers (IL-1ra, IL-2, IL-13, IL-15, IFN-γ, IP-10 and MCP-1) distinguished both the active TB and LTBI group from the QFT negative controls. IL-1ra, IL-2 and IP-10 also differentiated the QFT borderline group from the controls, and these three markers classified the majority of the study participants in the QFT borderline group in accordance with the QFT test.

Previous studies of alternative biomarkers from analyses of QFT supernatants [8–11] have found that unstimulated or TB antigen stimulated levels of various markers (EGF, MIP-1β, TGF-α, IL-1α, sCD40L, VEGF, IFN-α2, IL-1ra, IP-10, IL-2, IL-15 and MCP-1) may have potential for differentiating between active TB and LTBI. However, a review by Chegou *et al* concludes that there is no clear pattern of markers that are able to differentiate between the various stages of TB infection [13]. In support of this we also did not find any marker that was able to differentiate between active TB and LTBI when the background corrected TB antigen stimulated levels were analysed. However, in the unstimulated Nil supernatants, the levels of IL-1b, IL-1ra, IL-9 and IL17a were significantly lower in the active TB group compared with the LTBI group. This confirms studies that report that unstimulated levels of IL-1ra differentiate between active and latent TB in both children and adults [10,12].

In serial TB screening, relative high rates of conversions and reversions of QFT tests, especially around the diagnostic cut off, complicate the interpretation of IGRA results [2,3, 14]. A large variation in both these rates has been reported from different studies of HCW in TB low endemic countries (reversion rates 22–71% and conversion rates 1–14%) [2]. This was also seen in a cohort of health care students from a high TB incidence setting in which the variation could not be explained by occupational exposure [15]. Likewise, discordant IGRA results have been found in repeated testing of HIV-infected individuals living in TB low-endemic countries [4,5]. Pullar *et al* reported QFT reversions rates in HIV patients with untreated LTBI and in those receiving preventive TB therapy of 44% and 23%, respectively, during two years of follow-up as well as a conversion rate of 7% in the TB negative control group despite no known new TB exposure [4]. Finally, Metacalfe *et al* show that there is substantial variability in QFT responses when the test is repeated in the same patient sample [6]. In studies performed in TB low-endemic countries, the relative high conversion rates have been considered a result of false positive conversions. Thus, a borderline zone for QFT from 0.20–0.70 IU/mL has been suggested in the screening of HCW [2]. Altogether, these data highlights the need for additional markers to better discriminate between truly TB infected and uninfected individuals in order to identify those that should be offered preventive TB therapy.

In this study, the background corrected TB antigen stimulated levels of three markers, IP-10, IL-1ra and IL-2, differentiated the QFT borderline group from the QFT negative controls. ROC curve analyses for differentiation between these two groups showed that IP-10 had the highest AUC (0.92) and a sensitivity and specificity of 82 and 88% respectively, whereas IL-1ra had better sensitivity (100%) and IL-2 better specificity (94%). Based on the cut off levels found by the ROC curve analyses, IP-10, IL-1ra and IL-2 classified most of the study subjects as TB infected or not-TB infected in accordance with the QFT test. However, a few subjects in the active TB group were wrongly classified as not-TB infected by IL-2 and IL-1ra whereas some of the QFT negative subjects were classified as TB infected by the study markers. IP-10 had 100% sensitivity for active TB and classified two subjects in the QFT borderline zone as not TB. It is of interest that four of seven subjects in the QFT high group classified as not-TB by one or several of the three aforementioned markers had rather low QFT test results in the range of 0.70–1.0 IU/mL. Altogether this may indicate that a broader QFT borderline zone is more appropriate or at least that a positive QFT test in the lower range of the assay must be interpreted with caution. Still, the cut-offs from these limited ROC analyses can not at this stage be used as diagnostic tools in clinical practice as the data need to be validated in larger prospective studies.

IL-1ra, IL-2 and IP-10 have previously been suggested as diagnostic biomarkers for TB infection and IP-10 is the most extensively studied [7,13]. Although IP-10 is mainly secreted by antigen presenting cells (APCs), secretion is initiated by T cell recognition of specific peptides presented by APCs, and mainly driven by T cell derived IFN-γ. In accordance with our results, Ruhwald *et al* found that TB stimulated levels of IL-1-ra were significantly higher in patients with active TB compared with unexposed controls [16]. Frahm *et al* found the same pattern when comparing TB infected (active and LTBI combined) with uninfected subjects, and also a tendency of lower levels in LTBI versus active TB infection [11]. IP-10 and IL-2 offered comparable or even better sensitivity than IFN-γ for detection of patients with active TB [17–19] on support of our data. It has also been reported that the performance of IL-2 is similar to QFT in TB exposed individuals [19,20]. Whereas IP-10 has been equal to QFT in some studies [21,22] others have identified a higher number of infected contacts defined by IP-10 [19,20]. In our study, IL-1ra, IL-2 and IP-10 classified four, one and two, respectively, of the 16 QFT negative subjects as LTBI. To our knowledge, no studies of the variability of the alternative biomarkers in serial testing have been performed.

Previous studies show that unstimulated plasma levels of IP-10 distinguish between active and latent TB cases/household contacts [23,24]. We have also recently demonstrated that plasma IP-10 distinguished active TB from LTBI irrespective of HIV-infection and declined during anti-TB chemotherapy [25]. In support of our data, Chegou *et al* also found that the IP-10 Nil, but not TBAg-Nil level, in QFT supernatants distinguished between active TB and LTBI in children [10]. However, in the present study and a previous study in adults [9], neither the Nil nor the TBAg-Nil level of IP-10 in QFT supernatants were able to differentiate between these stages of infection. This discrepancy could be due to differences in the handling of the samples, the method of analysis and in the studied population.

There are some limitations to our study. Firstly, as there is no gold standard for diagnosing LTBI, the diagnostic accuracy of alternative markers is difficult to assess. In this study the diagnosis of LTBI was based on information of TB exposure, clinical examination excluding active TB and the QFT test result. Secondly, repeated testing of subjects with QFT test result in the borderline zone was not performed and we were therefore not able to examine the variability of IL-1ra, IL-2 and IP-10 in longitudinal samples. Thirdly, for some of the markers analysed, a considerable proportion of the study subjects in both the active TB and LTBI had TB stimulated and Nil levels that were above the upper detection level of the assay despite dilutions of the samples. Differences in these markers between the TB infection groups could therefore not be evaluated. When comparing the levels of these markers in TB groups with the controls, significant differences may not be detected because levels that were above the upper detection level were replaced by the highest value of the standard curve. The actual median levels in the active and LTBI group therefore may be higher than we were able to detect. Still, significant data presented in this study were all based on valid parameters. Finally, due to a small sample size increasing the risk of type 2 errors the data needs to be confirmed in larger prospective studies to determine whether the markers have reliable predictive capacity.

## Conclusions

Analysis of unstimulated levels of IL-1b, IL-1ra, IL-9 and IL-17a in QFT supernatants may help to discriminate active TB from LTBI whereas the respective TB antigen stimulated levels do no separate between the groups. Still, TB antigen stimulated IL-1ra, IL-2 and IP-10 levels differentiate the QFT borderline group from controls, supporting true TB infection in the majority of these patients. However, inconsistency was seen and further studies are needed to examine proper cut-offs and the variability of these markers in serial testing.
